# Clinical-based Cell Therapies for Heart Disease—Current and Future State

**DOI:** 10.5041/RMMJ.10401

**Published:** 2020-04-29

**Authors:** Darren Turner, Angela C. Rieger, Wayne Balkan, Joshua M. Hare

**Affiliations:** 1 Interdisciplinary Stem Cell Institute, University of Miami Miller School of Medicine, Miami, Florida, USA; 2 Department of Medicine, University of Miami Miller School of Medicine, Miami, Florida, USA

**Keywords:** Cell therapy, ischemic cardiomyopathy, non-ischemic cardiomyopathy, stem cells

## Abstract

Patients have an ongoing unmet need for effective therapies that reverse the cellular and functional damage associated with heart damage and disease. The discovery that ~1%–2% of adult cardiomyocytes turn over per year provided the impetus for treatments that stimulate endogenous repair mechanisms that augment this rate. Preclinical and clinical studies provide evidence that cell-based therapy meets these therapeutic criteria. Recent and ongoing studies are focused on determining which cell type(s) works best for specific patient population(s) and the mechanism(s) by which these cells promote repair. Here we review clinical and preclinical stem cell studies and anticipate future directions of regenerative medicine for heart disease.

## INTRODUCTION

The leading cause of death by non-communicable disease in the world is cardiovascular disease (CVD), and the American Heart Association estimates that over half of all Americans above the age of 40 suffer from CVD, much of it hypertension-related.^1^ While the number of Americans dying from CVD was decreasing, that trend began reversing in ~2012.^1^ Current treatments for CVD focus primarily on slowing disease progression or ameliorating pre-existing myocardial damage; however, the field lacks interventions that fundamentally reverse the progressive nature of CVD. Many patients with end-stage heart damage will require a heart transplant,^1^ yet there is a profound shortage of donors,^2^ illustrating the tremendous need for alternative/novel therapies.

One such approach is stem cell or cell-based therapy, a relatively new frontier in biomedical research that has sparked much debate and controversy in cardiovascular medicine.^3^,^4^ The heart was once thought to be incapable of regeneration, but the current consensus is that ~1%–2% of cardiomyocytes turn over each year with a rate that decreases with age. Cardiac remodeling has been characterized by a persistent inflammatory reaction after acute stress and during chronic pathologies,^5^ increased oxidative stress,^5^ myocyte apoptosis,^6^ imbalanced oxygen consumption, energy metabolism and extracellular matrix formation contributing to scar formation,^7^ endothelial dysfunction,^8^ and decreased capillary density and neovascularization.^9^ Stem cells and other cell-based therapies hold promise to counteract these effects and promote cardiac repair. Stem cells, strictly defined, possess the properties of both self-renewal and differentiation, whereas other cell-based approaches act through the transmission of factors that stimulate endogenous regenerative pathways. Current data support the idea that both approaches improve cardiac structure and function, and this implication of cardiac repair has spurred much excitement in the field.^10^ A current great debate is whether engraftment and differentiation of exogenously administered pluripotent stem cells is a requirement for a therapeutic response, with some investigators arguing that it is an essential requirement for a therapeutic response.^11^,^12^ This controversy is intensified by the observation that pluripotent stem cell therapies produce ventricular arrhythmias in preclinical, large-animal models,^11^,^13^,^14^ delaying clinical testing and the ability to compare these approaches with non-pluripotent cell-based therapies, which enjoy a substantial safety profile.

Clinical trials have assessed the safety and feasibility of cell-based therapy, largely testing culture-expanded cells from bone marrow, adipose tissue, or the heart itself. While initial studies demonstrated positive results, some trials have produced little or no functional improvements in cardiac performance. A majority of studies have focused on surrogate primary end points, such as changes in left ventricular ejection fraction (LVEF) and cardiac volumes, but in some studies only small improvements (5% on average) were seen, which has dampened enthusiasm toward the field.^15^–^17^ However, substantial efforts continue toward improving cell-based approaches for cardiac repair. Here, we will review clinical trials of cell-based therapy for heart disease and speculate on potential future directions of regenerative cardiovascular medicine.

## MECHANISMS OF ACTION

As mentioned above, a debate currently exists as to whether cell engraftment and differentiation is a requirement for a therapeutic response. Existing data suggest that functional and clinical responses can result from cell therapy using cell types that lack significant myocyte differentiation capacity.^18^–^20^ However, there is little evidence that stem cells engraft into the target tissue in the long term, suggesting a primarily paracrine mechanism of action.^21^,^22^ Indeed, secretions of exosomes, growth factors, cytokines, and metalloproteinases are mechanisms that contribute to the regenerative capacity of cells.^21^–^23^ Stem cells also interact with host cells via heterocellular coupling, wherein the cells communicate directly through gap junctions and tunneling nanotubes to transfer small molecules and mitochondria, respectively.^21^,^24^ However, a lack of a complete understanding of the mechanisms involved should not preclude clinical studies for evaluating efficacy.

**Figure 1:**
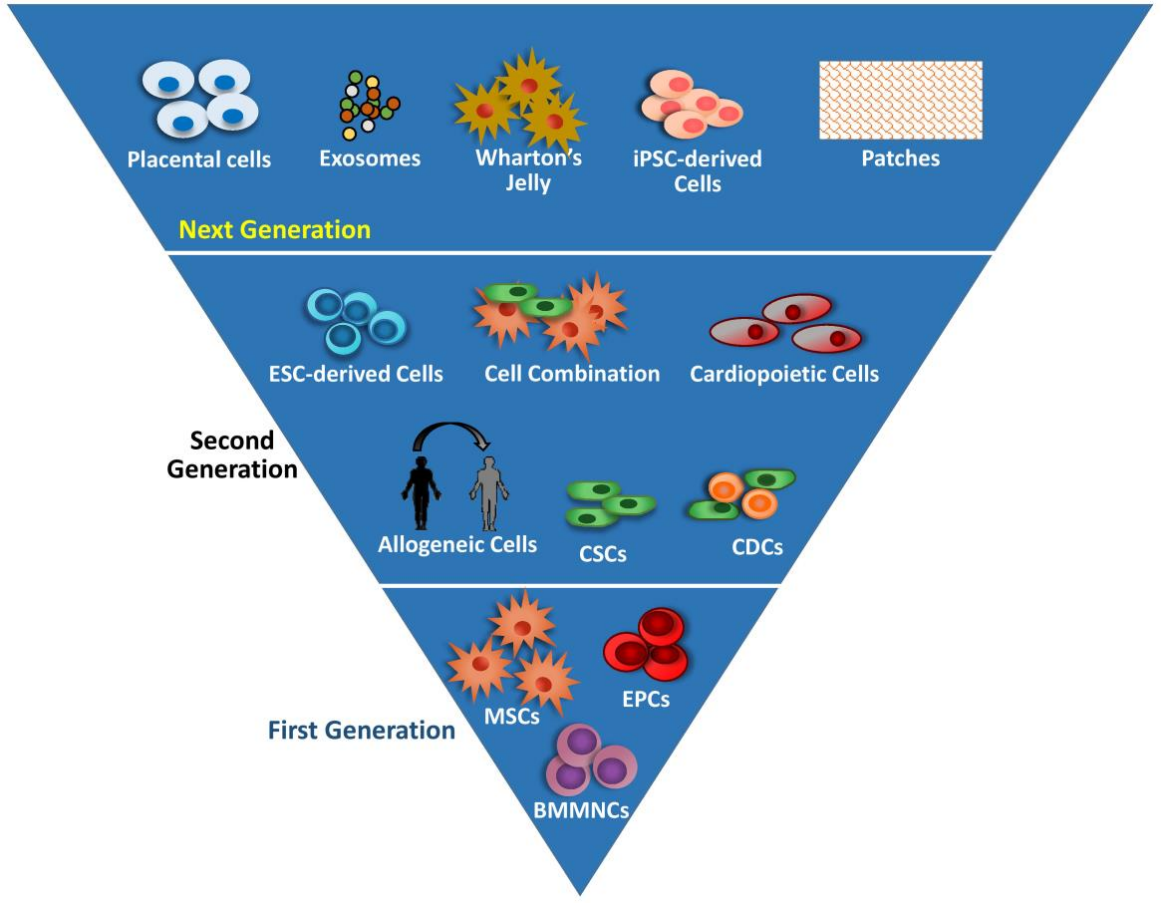
Advances in Cardio-regenerative Medicine First-generation stem cell therapy utilized heterogeneous populations of cells, such as BMMNCs and more purified MSCs and EPCs isolated from either bone marrow or blood. Second-generation stem cells include purified cardiac cell populations such as c-kit^+^ CSCs and CDCs, ESC-derived cells, allogeneic cells, cardiopoietic cells, and combinations of stem cells. Proposed next-generation approaches will utilize placenta and umbilical cord (Wharton’s jelly) cells, iPSC-derived cells, stem cell-derived exosomes, and cell-containing patches. ADRC, adipose-derived regenerative cell; BMMNC, bone marrow mononuclear cell; CDC, cardiosphere-derived cell; CSC, cardiac stem cell; ESC, embryonic stem cell; iPSC, induced pluripotent stem cell; MSC, mesenchymal stem cell. Adapted from Figure 3 of Banerjee et al.,^25^ used with permission. The Creative Commons license does not apply to this content. Use of the material in any format is prohibited without written permission from the publisher, Wolters Kluwer Health, Inc. Please contact permissions@lww.com for further information.

Initial or first-generation stem cells were/are derived from adult tissues, such as those isolated by bone marrow aspiration, and comprise either mixtures of different progenitor cell types, such as unfractionated bone marrow-derived mononuclear cells (BMMNCs), a heterogeneous population of stem cells, or more pure stem cell populations, many of which were isolated from BMMNCs (Figure 1). This latter group of cells includes hematopoietic stem cells (HSCs), mesenchymal stem cells (MSCs), endothelial progenitor cells (EPCs), and others.^26^ Mesenchymal stem cells, initially isolated from bone marrow, have been isolated from multiple tissues including adipose tissue, dental pulp, placenta, umbilical cord blood, and Wharton’s jelly.^27^,^28^ Mesenchymal stem cells exhibit properties important for a reparative cell, including immunomodulation^29^,^30^ and anti-fibrotic,^31^ proangiogenic, and anti-oxidative effects, all of which provide support for their being ideal candidates for treatment of cardiomyopathies. Furthermore, the lack of MHC class antigens confers immunoprivileged characteristics that make MSCs suitable for allogeneic therapy.^18^ Endothelial progenitor cells are primarily bone marrow-derived circulating progenitor cells characterized by the surface markers CD34 and CD133.^26^ Endothelial progenitor cell characteristics associated with their regenerative capacity include migration to injured areas to restore the endothelial niche, their proangiogenic properties, and ability to improve endothelial function.^32^

Second-generation cell therapies include cardiac-committed progenitor cells, that are lineage-directed by either genetic or secondary modifications to acquire a specific phenotype, or pluripotent stem cells (PSCs). Cardiac-committed progenitor cells include c-kit^+^ cardiac stem cells (CSCs). These clonogenic and multipotent cells can differentiate under secondary injury, contributing to organ regeneration. *In vitro*, CSCs display strong paracrine signaling and multilineage transdifferentiation, making them suitable for cardiac regeneration.^33^ Cardiosphere-derived cells (CDCs) are a heterogeneous population of cells isolated from myocardial tissue; they comprise CSCs and support cells and are capable of forming self-adherent clusters *in vitro*.^34^,^35^ These cells exhibit multilineage as well as clonogenic characteristics. Cardiopoietic cells are lineage-directed MSCs treated with multiple growth factors to bolster their stemness potential and differentiation.^36^

The PSCs (embryonic stem cells [ESCs] and induced pluripotent stem cells [iPSCs]) have the greatest multilineage capabilities^37^–^39^; however, the risk of teratoma formation requires that these cells first undergo lineage-directed differentiation prior to transplantation.^40^,^41^ Additional post-transplantation concerns include the risk of arrhythmias^11^,^13^,^14^ and rejection by the recipient. In preclinical studies these cells have demonstrated variable effects on the restoration of cardiac function.^11^,^13^,^14^

Third-generation therapy includes genetic reprogramming, exosomes, microRNA (miRNA), and the use of biomaterials to enhance the differentiation and regenerative capabilities of the cells.^40^ Exosomes are extracellular bilayer membrane vesicles that contain a diverse collection of proteins, lipids, and mRNAs/miRNAs and are secreted by a multitude of cell types.^42^ The exosomes secreted by iPSCs, ESCs, MSCs, and CDCs have different profiles,^43^,^44^ which ultimately physiologically manifest as increased self-renewal or expansion. Moreover, there is a growing body of evidence that exosome secretion is an important mode of cardiac cell communication.^21^,^22^

### Route of Delivery

**Figure 2:**
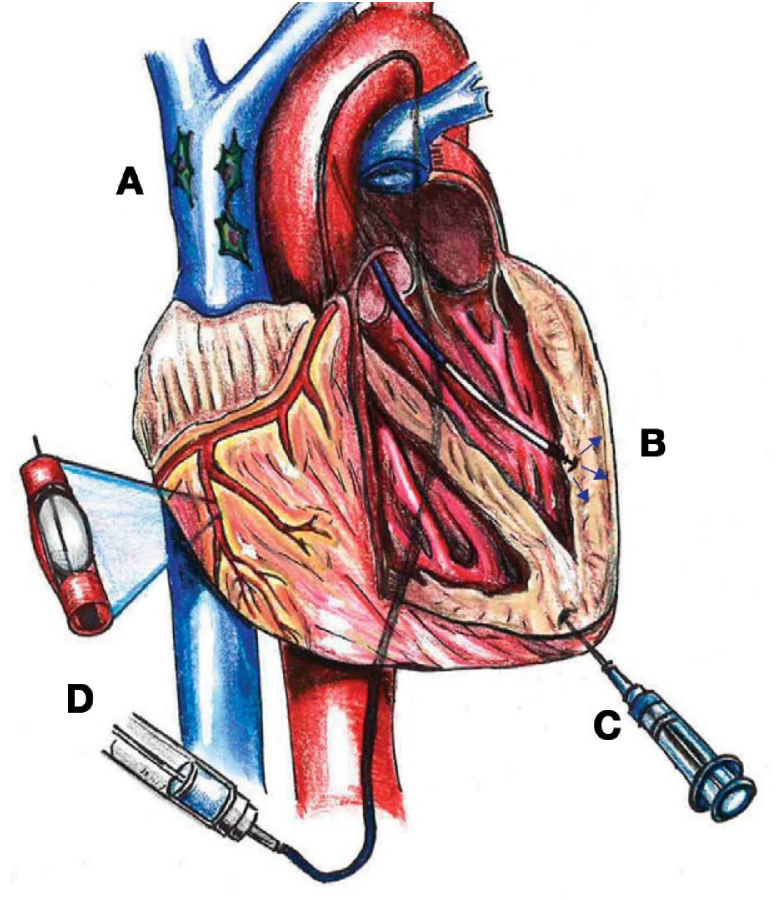
Various Approaches for Stem Cell Delivery to the Heart **A:** Intravenous delivery (peripheral veins not shown). **B:** Transendocardial stem cell injection (TESI) via catheter. **C:** Epicardial injection. **D:** Intracoronary infusion via catheter. From Figure 10 of Golpanian et al.^18^ with permission of the American Physiological Society.

Several factors contribute to the success of stem cell therapy. One of the most significant factors is the route of delivery^45^,^46^; yet there is no consensus on the best route. There are four primary methods of administration that are clinically practical, and each has its own advantages and disadvantages (Figure 2). For instance, although intracoronary delivery may cause poor cell retention in the heart, it carries the benefit of minimal inflammation.^47^ Transendocardial stem cell injections (TESI) are a minimally invasive technique where stem cells are injected directly into the myocardium through the endocardium. This procedure carries a small risk of perforation and arrhythmias; however, the retention of the cells is higher compared to other methods and in certain pathologies has shown greater effectiveness.^45^ Intravenous delivery of stem cells is the least invasive route and takes advantage of physiological attraction signals which induce cellular homing to the site of injury.^48^ With intravenous administration, there are concerns of poor implantation and retention. Unfortunately, very few studies have directly compared the therapeutic difference between routes of delivery.^49^ A meta-analysis of preclinical studies in models of acute myocardial infarction (AMI) by Kanelidis et al.^45^ concluded that TESI was associated with improved efficacy over intracoronary delivery. Additional preclinical and clinical studies are needed to establish an optimal route of delivery, and the most efficacious route may be cell-type dependent.

### Acute Myocardial Infarction

Bone-marrow derived mononuclear stem cells (BMMNCs) were some of the earliest cell types used in regenerative medicine to treat AMI.^50^,^51^ These cells are typically harvested from bone marrow and contain a heterogeneous group of cells including HSCs, MSCs, and EPCs.^52^ Because these cells can be obtained from bone marrow aspiration and do not require extensive expansion, BMMNCs are ideal for use in the setting of AMI and have been assessed in over 100 studies.^53^ Clinical trials involving BMMNCs were first conducted in the setting of AMI.

One of the first clinical trials testing therapeutic efficacy of BMMNCs was TOPCARE-AMI (Transplantation of Progenitor Cells and Regeneration Enhancement in Acute Myocardial Infarction).^54^ This study evaluated BMMNCs delivered an average of 5 days after an AMI. The trial reported a significantly increased LVEF and reduced scar size. Two years later, the BOOST (Bone Marrow Transfer to Enhance ST-Elevation Infarct Regeneration)^55^ trial showed similar promising results. It should be noted that the TOPCARE-AMI trial did not include a control group and the BOOST trial was open-label, since the control patients did not receive additional procedures, only standard care, compared to cell-treated patients. Another trial, LEUVEN-AMI, also reported improved LVEF after BMMNC infusion therapy.^56^ The REPAIR-AMI (The Reinfusion of Enriched Progenitor Cells and Infarct Remodeling in Acute Myocardial Infarction)^57^ trial is the largest phase III, double-blinded, placebo-controlled clinical trial to date and was conducted to assess the efficacy of BMMNCs. In this study, patients in the cell-treated arm had a significantly improved LVEF (5.5% in BMMNC group) compared to placebo (3.0%). After 1 year, death, myocardial infarction (MI), and the need for revascularization were lower in the BMMNC group.

Although the initial studies were exciting, many subsequent studies demonstrated, at best, inconclusive results. The ASTAMI (the Autologous Stem-Cell Transplantation in Acute Myocardial Infarction)^58^ trial, undertaken in 2006, demonstrated that after a 6-month follow-up, patients receiving cell treatment 6 days post-AMI showed no significant difference in LVEF or scar size compared to patients administered placebo. The multicenter, double-blinded, placebo-controlled TIME (Use of Adult Autologous Stem Cells in Treating People Who Have Had a Heart Attack)^59^ and LateTIME (Use of Adult Autologous Stem Cells in Treating People 2 to 3 Weeks After Having a Heart Attack)^60^ trials found no improvements in LVEF, left ventricular volumes, or wall motion as measured by cardiac magnetic resonance imaging between BMMNC and placebo groups. The SWISS AMI (Swiss Multicenter Intracoronary Stem Cells Study in Acute Myocardial Infarction)^61^ trial was a multicentered, open-labeled clinical trial that treated patients with BMMNCs either 5–7 days or 3–4 weeks after AMI. Neither group improved left ventricular function or scar size at 12 months; however, there was a high drop-out rate in this study. Finally, the repeat BOOST-2 trial^62^ was unable to replicate the results of the original study.

Preclinical data showed that a subpopulation of BMMNCs that were CD34^+^ could be a more suitable cell for AMI because of their angiogenic capacity.^63^ As such, the PreSERVE-AMI (NBS10 Versus Placebo Post ST Segment Elevation Myocardial Infarction)^64^ trial, the largest trial of stem cells for AMI in the United States, was conducted. This trial failed to show improvement in LVEF or resting myocardial perfusion; however, tertiary analyses demonstrated a significant association between change in LVEF and cell dose after adjusting for total ischemic time.^64^

The growing evidence of MSCs playing a key role in cardiac repair encouraged researchers to investigate their therapeutic efficacy in clinical trials.^65^ The effects of MSCs are the result of the secretion of cytokines, trophic factors, and matrix metalloproteinases which modulate the extracellular matrix and reduce infarct size and fibrosis.^31^ Compared to BMMNCs, human (h) MSCs are more efficacious in the setting of AMI. Hare et al.^65^ reported that precultured allogeneic hMSCs administered intravenously are safe, reduced episodes of ventricular tachycardia, and improved LVEF (6.7% greater than baseline). Another trial, WJ-MSC-AMI (Intracoronary Human Wharton’s Jelly-derived Mesenchymal Stem Cells Transfer in Patients with Acute Myocardial Infarction),^66^ demonstrated that MSCs derived from human umbilical cords increased LVEF (7.8%± 0.9% versus 2.8%±1.2%) and decreased end systolic volume (ESV) and end diastolic volume (EDV). There is also an ongoing trial, AMICI (Safety Study of Allogeneic Mesenchymal Precursor Cell Infusion in Myocardial Infarction), which is a phase II trial examining intracoronary delivery of mesenchymal precursor cells (NCT01781390).

Allogeneic CSCs have also been tested for therapeutic efficacy in the phase I/II, randomized, double-blind, placebo-controlled CAREMI trial (Cardiac Stem Cells in Patients with Acute Myocardial Infarction).^67^ Cardiac stem cells did not significantly improve scar size, left ventricular volumes, LVEF, or regional wall motion after 1 year of follow-up.

## CHRONIC ISCHEMIC CARDIOMYOPATHY

Despite advances in interventional care for AMI, patients frequently go on to develop chronic ischemic cardiomyopathy (ICM). The growing evidence of efficacy of stem cell treatment in AMI inspired researchers to begin experiments and clinical trials investigating stem cell therapy in chronic ICM. A major paradigm of treatment for ICM is the attenuation of left ventricular enlargement. Compared to mainstream treatment, regenerative medicine seeks to restore normal function, potentially being curative rather than palliative. As ICM is a chronic condition, researchers can utilize and cultivate a variety of cell types in an effort to maximize therapeutic effects.

There are significantly fewer studies carried out with BMMNCs in the setting of ICM. The first study exploring the effects of BMMNCs on patients with ICM was carried out by Perin et al.^68^ In this prospective, non-randomized, open-labeled study, BMMNCs were delivered via TESI. An evaluation performed 4 months later concluded that LVEF significantly increased from a baseline of 20% to 29%, which was accompanied by a reduction in ESV in treated patients. The TOPCARE-CHD (Transplantation of Progenitor Cells and Regeneration Enhancement in Chronic Postinfarction Heart Failure)^69^ trial showed small but similarly significant increases in LVEF with BMMNC treatment that correlated with reductions in N-terminal pro-brain natriuretic peptide (NT-proBNP). However, cell treatment did not reduce scar size compared to placebo. Subsequent studies were not able to replicate the positive effects on LVEF. The FOCUS-CCTRN (First Mononuclear Cells Injected in the United States conducted by the Cardiovascular Cell Therapy Research Network)^70^ trial, a phase II, randomized double-blind, placebo-controlled study, showed no significant difference in LVEF or infarct size in patients treated with BMMNCs.

The TAC-HFT (The Transendocardial Autologous Cells [hMSC or hBMC] in Ischemic Heart Failure Trial)^71^ was one of the earliest trials examining MSCs as a treatment for ICM. Importantly, it was the first trial to compare BMMNCs to MSCs head-to-head. This phase II randomized, placebo-controlled study failed to demonstrate improvements in LVEF or left ventricular volumes; however, both groups showed improvement in quality of life (QoL) as measured by the Minnesota Living with Heart Failure Questionnaire (MLHFQ) score. Additionally, the 6-minute walk distance (6MWD) improved only in the MSC group. Infarct size was reduced by 19% in the MSC group, whereas in the BMMNC and placebo groups it remained unchanged. The POSEIDON trial (Percutaneous Stem Cell Injection Delivery Effects on Neomyogenesis)^72^ study compared allogeneic to autologous MSCs and demonstrated that both MSC groups reduced scar size by ~33%, consistent with the TAC-HF trial. Of note, both the POSEIDON and TAC-HFT studies showed that QoL can be improved without concomitant improvements in LVEF. The MSC-HF trial^73^ assessed TESI of autologous MSCs and showed increased LVEF of 6.2% compared with placebo, as well as reduced left ventricular ESV. These increases were maintained at the 1-year follow-up while myocardial mass was greater than at 6 months.^74^ There was a correlation between cell dose and improvements.^74^ The randomized, double-blinded TRIDENT trial^75^ examined dose-dependence of allogeneic MSCs. In this study, the 100 million cell dose improved absolute LVEF by 3.6% compared to no change in the 20 million cell dose group after 12 months. Additional, larger clinical trials are needed.

A more recent approach is treatment using combinations of stem cells, which may provide greater therapeutic efficacy than a single cells type, as was observed with MSCs+CSCs in preclinical studies in porcine models of ICM.^76^–^78^ These porcine studies formed the basis of the ongoing phase II CONCERT-HF trial (Combination of Mesenchymal and C-kit^+^ Cardiac Stem Cells as Regenerative Therapy for Heart Failure),^79^ which is assessing if the combination of MSCs plus CSCs provides greater cardiac repair in humans than either cell type alone. Other clinical^80^ and preclinical^77^,^81^ studies have also demonstrated a positive effect of combination stem cell therapy.

Cardiospheres were first described after a population of cells isolated from subcultures of atrial or ventricular biopsy specimens were shown to be able to differentiate into cardiomyocytes, endothelial cells, and smooth muscle cells.^34^ Cardiospheres contain a heterogeneous mixture of cell types including cells that express endothelial (KDR [human]/flk-1 [mouse], CD31), stem cell (CD34, c-kit, Sca-1), and mesenchymal (CD105, CD90) cell surface markers.^34^ However, the specific cell type contributing to cardiac functioning and remodeling is unknown.^82^ Promising preclinical work provided the basis for a phase I, randomized trial, CADUCEUS (Cardiosphere-Derived Autologous Stem Cells to Reverse Ventricular Dysfunction),^83^ in patients with ICM. At 1.5–3 months after MI, 17 patients were administered intracoronary injections of autologous CDCs (98% of cells given were CD105-positive). Although scar size was reduced by 42% in the treatment arm, CDC therapy failed to increase LVEF, reduce left ventricular volumes, or improve QoL as measured by MLHFQ. The ALLSTAR (Allogeneic Heart Stem Cells to Achieve Myocardial Regeneration)^84^ trial using CDCs had to be suspended by the Data Safety Monitoring Board because the study failed to meet the primary end point. Given the heterogeneous nature of this cell preparation, it may be difficult to identify which cell type(s) truly contributes to any beneficial effects.

Cardiopoietic stem cells (CPSCs) are MSCs that are manipulated *ex vivo* to undergo cardiopoiesis in order to enhance their cardio-reparative functionality.^85^,^86^ The randomized, double-blinded, placebo-controlled CHART-1 (Congestive Heart Failure Cardiopoietic Regenerative Therapy)^87^ trial was conducted to ascertain the safety and therapeutic value of CPSCs in patients with ICM. The primary efficacy end point of a Finkelstein–Schoenfeld hierarchical composite (mortality, worsening heart failure, MLHFQ, 6MWD, LVEF, ESV) at 39 weeks was not achieved.

## NON-ISCHEMIC DILATED CARDIOMYOPATHY

Non-ischemic dilated cardiomyopathy (NIDCM) is the leading cause of death among heart transplant recipients.^88^ As with ICM, BMMNCs were the first cell type to be tested in the setting of NIDCM. In the TOPCARE-DCM (Transplantation of Progenitor Cells and Recovery of Left Ventricular Function in Patients with Non-Ischemic Dilatative Cardiomyopathy)^89^ trial, patients showed improvements in LVEF, regional wall motion at 3 months after treatment, and decreased NT-proBNP levels at 1-year follow-up. Similarly, the ABCD (Autologous Bone Marrow Cells in Dilated Cardiomyopathy)^90^ trial found positive results, including QoL parameters, which conflicted with the MiHeart,^91^ a multicenter, randomized, double-blind clinical trial that evaluated intracoronary delivery of BMMNCs and showed no significant changes in LVEF and left ventricular volumes.

**Figure 3:**
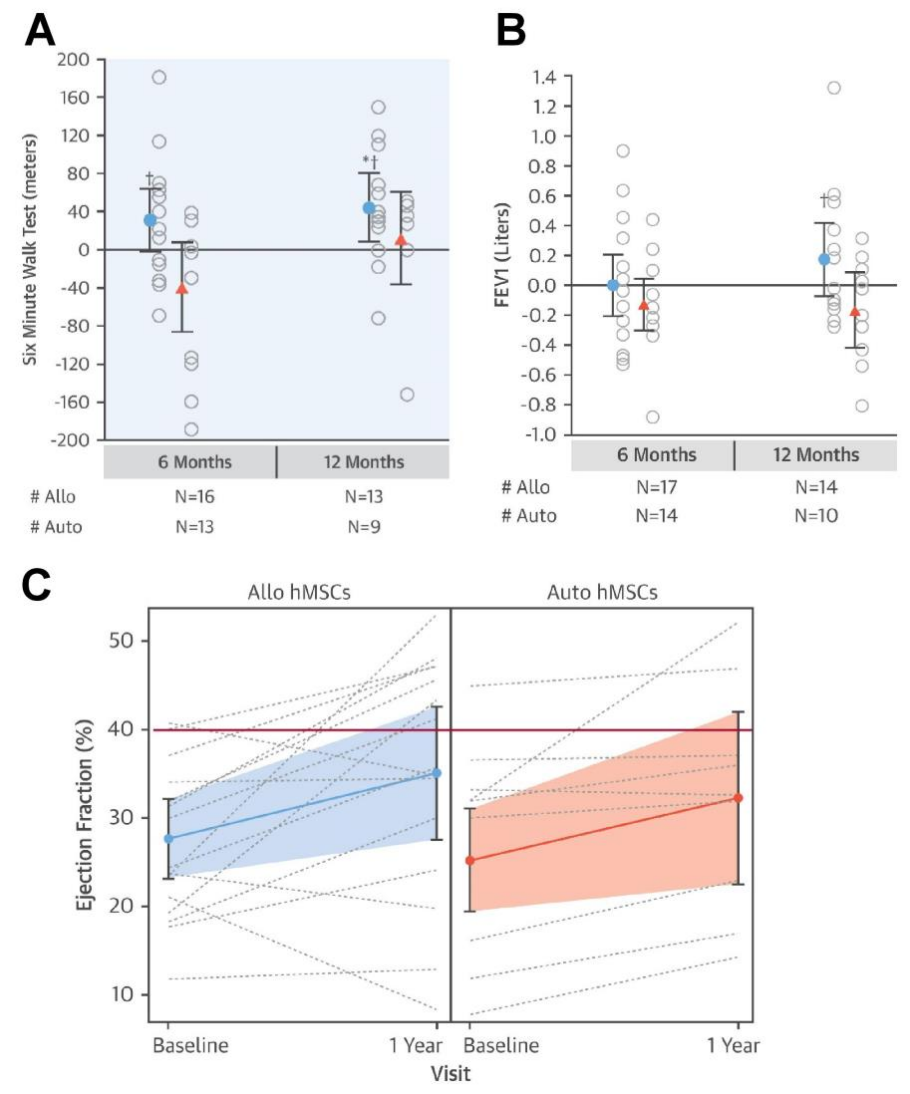
Differences in the Therapeutic Efficacy of Allogeneic hMSC and Autologous hMSC The POSEIDON-DCM^95^ study demonstrated differences in the therapeutic efficacy of allogeneic (Allo, blue) hMSC, and autologous (Auto, red) hMSC. **A:** Change from baseline in the 6-minute walk distance (6MWD). **B:** Change from baseline in forced expiratory volume in 1 second. **C:** Individual patient response in ejection fraction; shaded areas are 95% CI. From Hare et al.^95^ [CC BY-NC-ND 4.0].

**Figure 4:**
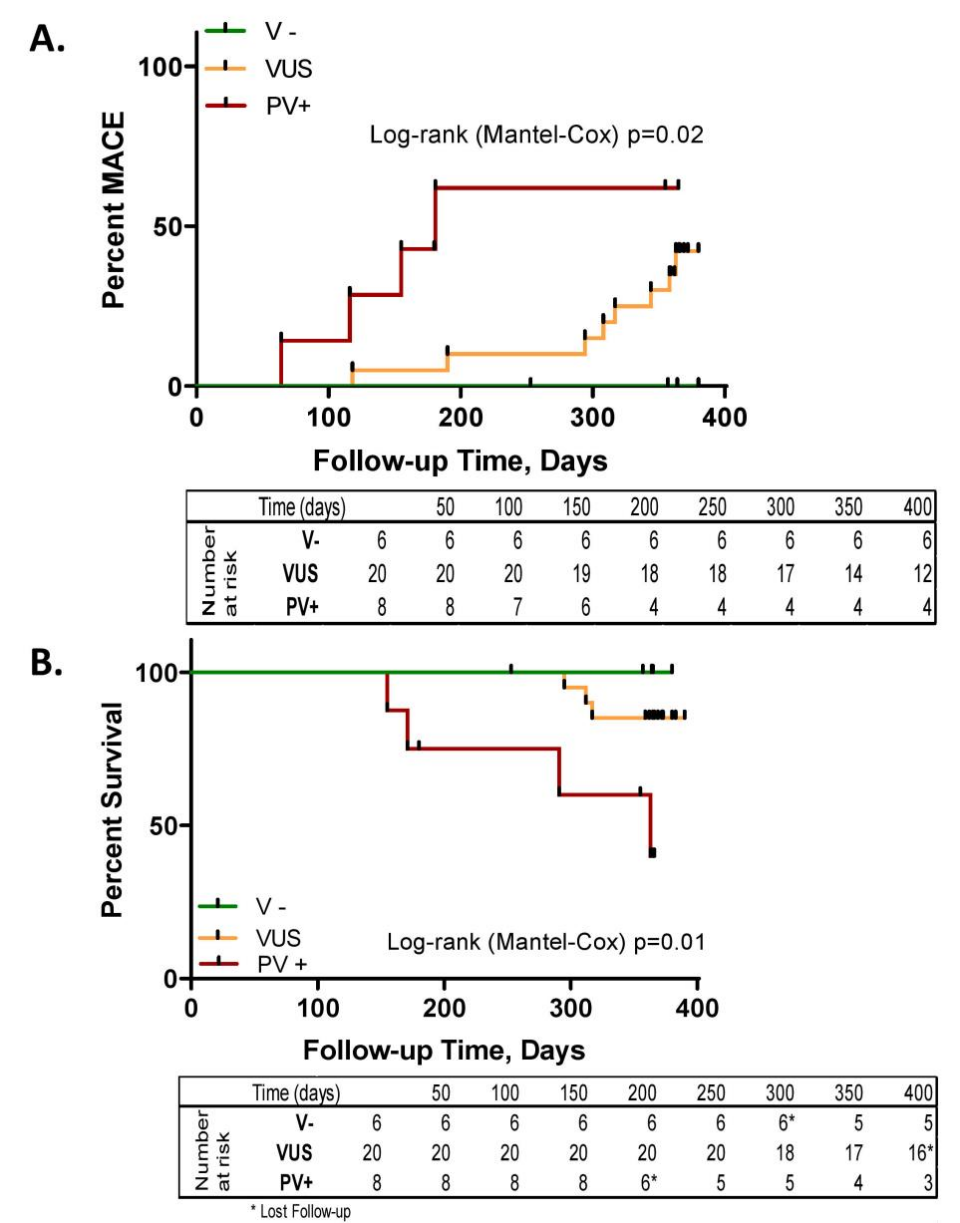
Genetic Variation Affects Major Adverse Clinical Events (MACE) and Survival in Response to Delivery of MSCs POSEIDON-DCM patients negative for pathologic genetic variants (V−, green) had fewer MACE **(A)** and greater survival **(B)** than patients with variants of uncertain significance (VUS, orange) or pathologic/likely pathologic variants (PV+, red). Overall, PV+ patients had a substantial increase in death, transplant, or left ventricular assist device (LVAD) risk by 1-year follow-up. From Figure 3, Rieger et al.^96^ [CC BY-NC-ND 4.0].

Compared to ICM, NIDCM has a more significant immunologic component.^92^ As such, MSC ther-apy could prove beneficial due to its immunomodulatory, reverse remodeling, and regenerative properties.^93^,^94^ The POSEIDON-DCM trial (Percutaneous Stem Cells Injection Delivery Effects on Neomyogenesis in Dilated Cardiomyopathy)^25^ randomly allocated 37 patients with idiopathic NIDCM to receive TESI of allogeneic or autologous MSCs. Functional parameters and LVEF increased significantly only in the allogeneic group (Figure 3). Of note, LVEF increases were not accompanied by reductions in left ventricular volumes, suggesting that reverse remodeling is not the primary means by which cardiac function is improved. Incidence of major adverse cardiac events and hospitalization rate was also significantly lower in the allogeneic group.^25^ Moreover, treatment with allogenic MSCs significantly increased QoL and functional capacity. Both treatment arms noted significantly decreased systemic tumor necrosis factor (TNF)-α levels. The POSEIDON-DCM trial also demonstrated that patients lacking a pathologic genetic variant responded better to cell therapy (Figure 4).^96^ However, this study lacked a control group, and this approach should be further investigated in a larger study. A study by Vertelov et al. observed that ischemia-tolerant MSCs, i.e. hMSCs cultured under hypoxic conditions, are more therapeutically efficacious than hMSCs grown in normoxia.^97^ To ascertain this effect *in vivo*, Butler et al.^98^ conducted a pilot study in which bone marrow-derived MSCs isolated from healthy donors were grown under hypoxic conditions and subsequently administered to 22 patients. Although no improvements in left ventricle anatomy or function were noted, QoL and 6MWD scores improved significantly in the treatment arm.

Interestingly, the administration of CD34^+^ cells demonstrated consistent improvements in LVEF,^99^ 6MWD, brain natriuretic peptide (BNP) levels, as well as survival at 1 and 5 years post-treatment.^99^ The comparison between intra-coronary and TESI delivery demonstrated that TESI produced higher engraftment and therapeutic efficacy.^49^ Importantly, a subset of patients with NIDCM and diabetes mellitus did not respond similarly to the non-diabetic population, which had an improvement in LVEF.^100^ These studies demonstrate that specific subpopulations of patients respond to a greater or lesser extent to the same therapy, illustrating the importance of adequately assessing the profile of patients, the cell(s) to be delivered, and the route of delivery.

## OTHER POTENTIAL SOURCES OF CELLS FOR CARDIAC REPAIR

### Pluripotent Stem Cells

While many stem cells have been tested for their cardio-reparative capacity, ESCs and iPSCs have yet to be thoroughly assessed in clinical trials. As mentioned above, these cells have the greatest multilineage capability, but also some of the highest potential risks.^37^–^39^,^101^

### Embryonic Stem Cells

Embryonic stem cells are immortal, pluripotent cells derived from the inner cell mass of the pre-implantation embryo, that are propagated *ex vivo*.^102^,^103^ However, the risk of teratoma formation requires that these cells first undergo lineage-directed differentiation prior to transplantation.^40^,^41^ Additional concerns after transplantation are the risk of arrhythmias and rejection by the recipient.

**Figure 5:**
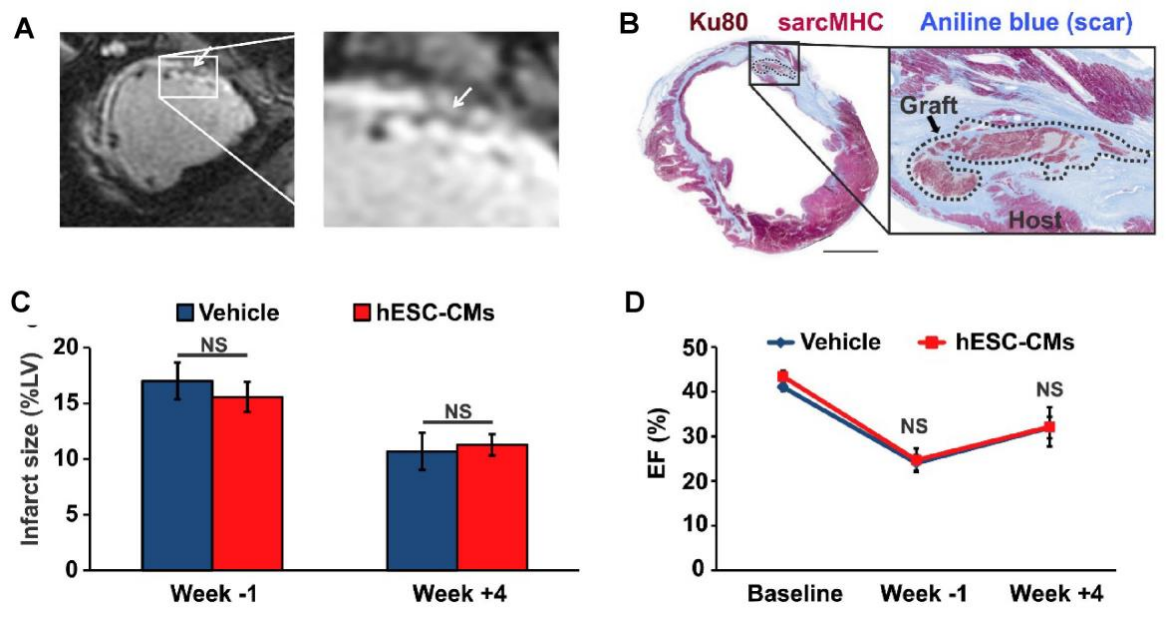
In a Porcine Model of MI, hESC-CMs Demonstrated Engraftment but no Functional or Structural Improvements Islands of engrafted cells were seen in immunosuppressed pigs receiving hESC-CMs by magnetic resonance imaging (**A)** and immunostaining **(B)**. The hESC-CM-injected pigs demonstrate no reduction in scar size **(C)** or improvement in LVEF **(D)**. Adapted from Romagnuolo et al.^14^ [CC BY 4.0].

Two preclinical studies involved the intramyocardial administration of human ESC-derived cardiomyocytes (hESC-CMs) into non-human primates following ischemia/reperfusion injury.^11^,^13^ Pigtail macaques were kept immunosuppressed and administered 1×10^9^ hESC-CMs 2 weeks post-MI^11^ or 750×10^8^ hESC-CMs 4 weeks post-MI.^13^ The hearts exhibited islands of engrafted hESC-CMs, but there was no reduction of infarct size, and non-lethal ventricular arrhythmias were seen in all animals.^11^,^13^ A similar study was conducted using 1×10^9^ hESC-CMs in a porcine model of MI. Similar to the macaque studies, the immunosuppressed pigs had islands of engrafted hESC-CMs but no cardiac functional or structural improvements (Figure 5).^14^ These results suggest that further preclinical studies are needed to optimize the therapeutic effects of hESC-CMs. However, Menasche et al. demonstrated that hESC-derived cardiac progenitor cells embedded into a fibrin scaffold are safe in a patient with severe heart failure.^41^ Cell therapy did not cause complications, and after 3 months the patient showed improvements in cardiac function. Importantly, the European clinical study, ESCORT, in which hESC-derived cardiac progenitors were transplanted within a fibrin patch into heart failure patients (*n*=6), showed safety and efficacy.^41^ One patient died early post-operation from treatment-unrelated comorbidities. The other 5 patients showed no evidence of significant adverse effects (SAEs), and they improved symptomatically with an improved wall motion of the cell-treated segments.

### Induced Pluripotent Stem Cells

Due to the ethical concerns of harvesting ESCs, scientists have sought alternative methods to isolate multipotent stem cells. Takahashi and Yamanaka developed a novel protocol to generate pluripotency from murine somatic cell by integrating a variety of transcription factors into the cell’s genome via retroviral transduction.^104^ This technique was then applied to human somatic cells.^105^ Subsequent studies have demonstrated that these iPSCs have the capacity to differentiate into all three germ layers in addition to somatic cells, including cardiomyocytes and other cardiovascular cells.^106^,^107^ Furthermore, these cells could also aid in repair of heart valves and vessels.^108^ A major concern when using these pluripotent cells, as with ESCs, is tumorigenesis. However, this risk can be mitigated by isolating cells or cell lines that have undergone at least some differentiation.^109^ An initial clinical trial to evaluate safety and efficacy of a patch with 100 million reprogrammed iPSC cardiomyocytes was approved in Japan. Three patients with ICM were treated initially; a further 7–10 patients will be recruited and followed up over the period of 1 year.^110^ The Treating Heart Failure With hPSC-CMs (HEAL-CHF) Trial (NCT03763136) is an open-label study recruiting 5 patients to receive epicardial injection of allogeneic PSC-CMs. There are as yet no reports from either of these two studies. Continuing studies will have to investigate methods to maintain stable cell lines as well as address scalability for clinical grade production.^111^ Table 1 compares the efficacy of different cell types for increasing LVEF, and reducing EDV and scar size in clinical trials to date.

**Table 1:** Comparison of Cell Types.

Cell Type	Preclinical Animal Studies (*n*)		Completed Clinical Studies at ClinicalTrials.gov (*n*)			Change in Clinical Data							
						LVEF (%)			EDV (mL)			Change in Scar Size (%)	
	AMI	ICM	AMI	ICM	NIDCM	AMI	ICM	NIDCM	AMI	ICM	NIDCM	AMI	ICM
MSCs	335	133	4*	6†	2‡	6.0	5.7	8.0	N/A	−9.3	N/A	−6.2	−25.9
CPCs	182	87	0	1§	0	N/A	5.4	N/A	N/A	−12	N/A	N/A	−12
ESCs	3	0	0	1**	0	N/A	12.5	N/A	N/A	−14.5	N/A	N/A	N/A
iPSCs	3	0	0	0	0	N/A	N/A	N/A	N/A	N/A	N/A	N/A	N/A

* References 65, 66, 112, and 113.

† References 71–74, 75, 114, and 115.

‡ References 25 and 98

§ Reference 116.

** Reference 41. Cells delivered via patch not intramyocardial.

Initial search criteria: Heart or cardiac, cells, completed studies, with results (=148 results on clinicaltrials.gov). An advanced search of these results was performed for “acute myocardial infarction,” “ischemic cardiomyopathy,” or “non-ischemic dilated cardiomyopathy,” each with the intervention of: “mesenchymal stem cells” for MSCs; “cardiosphere-derived stem cells” and “cardiopoietic stem cells” for CPCs; “embryonic stem cells” for ESCs; or “induced pluripotent stem cells” for iPSCs.

N/A, data not available.

### Placental Stem Cells

The placenta is a novel source of potentially cardio-regenerative cells. Perinatal tissue is a rich source of a variety of stem cells that can be isolated from the amnion, chorion, umbilical cord (e.g. Wharton’s jelly) and the placental cotyledons from the fetal side and the decidua from the maternal side. Many of these cells display MSC-like characteristics, such as adherence to plastic and immunomodulation. Furthermore, *in vitro*, they inhibit cardiomyocyte apoptosis and are pro-angiogenic (reviewed by Bollini et al.^117^).

Cells isolated from the murine near-term placenta and expressing the Caudal-type homeobox-2 (Cdx2) were recently reported to form beating cardiomyocytes and vascular lineages *ex vivo*. Furthermore, these Cdx2^+^ cells homed to the injured heart and promoted cardiac repair when injected intravenously (1×10^6^ cells) post-MI in a mouse model. Three months post-injection, the cells were found integrated within the myocardium, primarily in the border zone, where they exhibited a cardiomyocyte morphology. Cell-treated hearts exhibited improved LVEF and stroke volume and reduced adverse remodeling compared to placebo-injected mice.^118^

### Tissue-specific MSCs

Most studies have assessed the therapeutic effects of bone marrow- and adipose tissue-derived MSCs. These cells can be isolated and expanded in large quantities while retaining their immunomodulatory characteristics,^119^ but the properties of these MSCs are influenced by their tissue of origin. For example, bone marrow-derived MSCs are highly proangiogenic^120^ and may be more immunosuppressive than adipose-derived MSCs.^121^,^122^ Mesenchymal stem cells have also been isolated from other tissues, including umbilical cord (Wharton’s jelly), amniotic fluid, peripheral blood, and the heart. Again, the tissue of origin appears to provide MSCs with characteristic properties^122^–^126^ and secretomes,^127^ and for therapeutic use it may be important to determine which MSC source is best for a specific patient.

## EXOSOMES/MICROVESICLES

Some studies suggest that exosomes have an (almost) equivalent therapeutic efficacy as intact cells.^128^–^130^ Other data also demonstrate that the therapeutic effect of cell therapy may not correlate with engraftment,^131^ supporting a paracrine mechanism. The discovery of these paracrine mechanisms of repair not only significantly challenges the notion of engraftment-dependent healing, but also opens another avenue of therapy delivery.^42^,^132^ Engineered exosomes with an ischemic myocardium-targeting peptide can enhance myocardial viability and reduce infarct size after MI in mouse models.^43^,^133^–^135^ Cell-free suspensions containing important reparative exosomes could be used instead of intact cells, avoiding some of the inherent issues associated with cells, such as *ex vivo* expansion, tumor formation, and immune rejection. Studies directly comparing the different approaches will provide guidance toward the most therapeutic approach.

## PATCHES/BIOMATERIALS: BIOENGINEERING IN STEM CELL THERAPY

Transplantation of viable cells into the harsh environment of necrotic myocardium remains a significant therapeutic challenge resulting in very poor cell retention.^136^,^137^ To combat this problem, tissue engineering approaches have designed biomaterials as cell retention mediums. These injectable biomaterials must perform many (often contradictory) functions. They must be biodegradable, biocompatible, provide mechanical support, be of appropriate dimension, allow for precise placement,^138^ improve cell survival, and promote tissue regeneration.^139^,^140^ These polymers can either be synthetic or naturally derived, each having their own advantages and disadvantages. Some polymers can even be specifically tailored to optimize cardiac repair,^141^ and 3D-printing has increased the available types of biomaterials, improving cell integration and vascularization.^142^ Preclinical studies have demonstrated improved cell viability and cardiac repair when used with human pluripotent stem cells and MSCs.^141^,^143^,^144^ While significant progress has been made, improving polymer compatibility and mechanical properties must occur before clinical studies can begin.

## FUTURE DIRECTIONS

Stem cell and cell-based therapy is still relatively new, and studies need to define the cell type/cell product, the frequency and route of stem cell injection, and the patient population most likely to respond. Recent preclinical studies show that the administration of a large number of exosomes often produces similar cardiac repair as cell injection,^145^,^146^ prompting the view that the cells are not needed. However, this equivalency is often dependent on the route of exosome administration and has only been demonstrated in the short term, while stem cell therapy has demonstrated long-term effects, despite poor stem cell retention and survival. Studies comparing the long-term effects of cells versus exosomes (or combination of the two) still need to be performed.

Other approaches toward optimizing stem cell therapy include assessing the effects of multiple rounds of injections. Tokita et al. demonstrated that three rounds of cardiac progenitor cell injections provided greater cardiac repair than a single injection of 3-fold more cells in a mouse model of ischemia.^147^ As mentioned above, the administration of a combination of stem cells is therapeutically synergistic, providing greater benefits than the individual cells in swine models of ICM,^74^–^76^ and this approach forms the basis of the CONCERT-HF clinical trial.^77^

**Figure 6:**
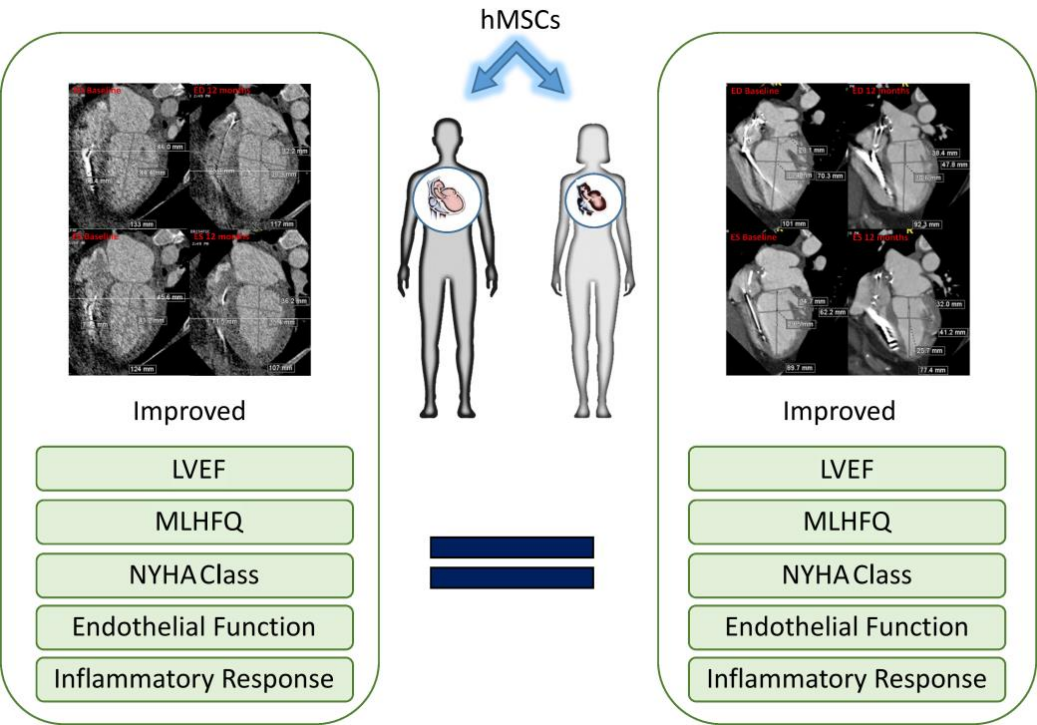
Effectiveness of MSCs Is Independent of Sex of the Patient Cell therapy improves cardiac function, functional parameters, endothelial function, and inflammatory response independently of the sex of the patient. LVEF, left ventricular ejection fraction; MLHFQ, Minnesota Living with Heart Failure Questionnaire; NYHA Class, New York Heart Association Functional Classification. Adapted from the graphical abstract in Florea et al.^148^; by permission of Oxford University Press.

Additionally, the choice of patient is important. While the CHART-1 study did not meet its primary end point, a subpopulation of patients responded well.^36^,^85^ Precision medicine approaches may also influence a patient’s responsiveness to stem cell therapy. As mentioned above, Rieger et al. recently showed that, in the POSEIDON-DCM trial, patients who did not have a specific genetic variant responded better to treatment.^96^ Furthermore, sex,^148^ age,^149^ and serum concentration of a variety of factors^150^ may play significant roles in a patient’s response and need to be taken into account when designing clinical trials (Figure 6).

Clinical trials will also need to be nimble and develop better ways to assess efficacy and increase study power by incorporating ongoing results as well as new information that becomes available after the trial commences. The DREAM-HF (Double-Blind Randomized Assessment of Clinical Events With Allogeneic Mesenchymal Precursor Cells in Advanced Heart Failure; NCT02032004) is such a trial. It is a phase III, randomized, placebo-controlled study assessing the safety and efficacy of mesenchymal precursor cells (MPCs) as immunotherapy in patients with advanced, chronic heart failure with reduced ejection fraction. The DREAM-HF trial uses patient enrichment strategies to establish a patient population with reduced heterogeneity (baseline disease enrichment), high targeted outcome event rate (prognostic enrichment) and higher likelihood to respond (predictive enrichment). Adaptive statistical models are also needed. The DREAM-HF trial uses a joint frailty model, which treats terminal and recurrent heart failure events differently and models correlations between recurrent and terminal events, which takes into account random, between-patient differences. Such innovative approaches will allow for smaller, yet more definitive trial designs.^151^ Along with CONCERT-HF, DREAM-HF is likely to report results in 2020, and together these trials will add substantially to the clinical and mechanistic data base of the potential of cell-based therapy for chronic heart failure.

## CONCLUSION

The past two decades have witnessed substantial translational efforts to develop cell-based therapies for heart disease. While many clinical trials have been conducted, testing several strategies, the field has yet to yield a clear understanding of the clinical application in this important area. Nonetheless, the studies conducted to date have laid a robust groundwork for ongoing new efforts—including phase III and larger-powered phase II studies, as well as major progress at the bench and in preclinical models in the application of pluripotent stem cells. With these ongoing avenues of research, the field is moving closer to yielding a successful strategy for addressing one of the largest unmet needs in modern medicine, that of chronic heart disease.

## Abbreviations

AMI: acute myocardial infarction
BMMNCs: bone marrow-derived mononuclear stem cells
BNP: brain natriuretic peptide
CDCs: cardiosphere-derived cells
CPSCs: cardiopoietic stem cells
CSCs: cardiac stem cells
CVD: cardiovascular disease
EDV: end diastolic volume
EPCs: endothelial progenitor cells
ESCs: embryonic stem cells
ESV: end systolic volume
HSCs: hematopoietic stem cells
iPSCs: induced pluripotent stem cells
ICM: ischemic cardiomyopathy
LVEF: left ventricular ejection fraction
MI: myocardial infarction
MSCs: mesenchymal stem cells
NIDCM: non-ischemic dilated cardiomyopathy
QoL: quality of life
6MWD: 6-minute walk distance
TESI: transendocardial stem cell injection
